# A GABAergic Dysfunction in the Olivary–Cerebellar–Brainstem Network May Cause Eye Oscillations and Body Tremor. II. Model Simulations of Saccadic Eye Oscillations

**DOI:** 10.3389/fneur.2017.00372

**Published:** 2017-08-04

**Authors:** Lance M. Optican, Elena Pretegiani

**Affiliations:** ^1^Laboratory of Sensorimotor Research, IRP, National Eye Institute, National Institutes of Health, Bethesda, MD, United States

**Keywords:** saccade, vermis, fastigial nuclei, inferior olive, omnipause neurons, eye movement, flutter, opsoclonus

## Abstract

Eye and body oscillations are shared features of several neurological diseases, yet their pathophysiology remains unclear. Recently, we published a report on two tennis players with a novel presentation of eye and body oscillations following self-administration of performance-enhancing substances. Opsoclonus/flutter and limb tremor were diagnosed in both patients. Common causes of opsoclonus/flutter were excluded. High-resolution eye movement recordings from one patient showed novel spindle-shaped, asymmetric saccadic oscillations (at ~3.6 Hz) and ocular tremor (~40–60 Hz). Based on these findings, we proposed that the oscillations are the result of increased GABA_A_ receptor sensitivity in a circuit involving the cerebellum (vermis and fastigial nuclei), the inferior olives, and the brainstem saccade premotor neurons (excitatory and inhibitory burst neurons, and omnipause neurons). We present a mathematical model of the saccadic system, showing that the proposed dysfunction in the network can reproduce the types of saccadic oscillations seen in these patients.

## Introduction

Oscillations of the head, body, limbs, or eyes characterize several neurological conditions. Nevertheless, their underlying mechanisms are not understood well enough to guide therapy (1). Oculo- and somatomotor systems are similarly organized, and some disorders involve both eye and body oscillations. The anatomy and physiology of the oculomotor system have been studied more than that of other systems. If we could understand ocular oscillations, it might provide insights into the pathophysiology of oscillatory dysfunctions of somatomotor circuits. Here, we present a mathematical model of the saccadic system that has sufficient anatomical and physiological detail to simulate saccadic eye oscillations in patients with opsoclonus. A model of ocular and limb tremor will be presented in a companion paper.

**Figure 1:**
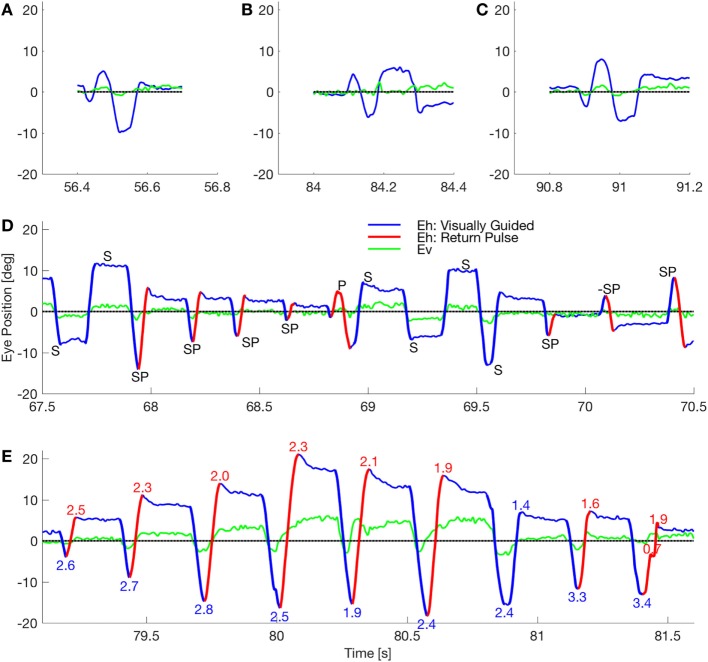
Waveforms in a patient with opsoclonus. **(A–C)** The subject made few of the sinusoidal movements that classically define opsoclonus. There were occasional movements with 0.5–1.5 cycles, but these three movements were the longest oscillations in a fixation record that lasted 156 s. **(D)** The subject mostly made macrosaccadic square-wave oscillations (marked S), back-to-back oscillations with no intersaccadic intervals (pulses, marked P), and a combined half-square wave and pulse waveform (square-pulse, marked SP). Note that all these waveforms occurred within a 3 s window. The direction of the square-pulse oscillation suddenly reversed at about 70.15 s (marked −SP). Finally, the duration of a square-wave half-cycle can vary (e.g., the short cycle at ~69.6 s), suggesting that there is a spectrum from opsoclonus to square-pulse to square-wave oscillations. **(E)** The subject made several series of square-pulse oscillations with an increasing gain followed by a decreasing gain (indicated by the numbers at the extreme positions). This type of movement is called a *spindle*. Square-wave spindles are common in cerebellar disease, but this is the first square-pulse spindle to be reported. By comparing the timing of the horizontal and vertical eye movements in these panels, it is evident that the macroscopic movements are coupled.

Previously, we studied two patients with a novel presentation of eye and body oscillations following self-administration of performance-enhancing substances (2). Opsoclonus consists of large saccadic oscillations lasting a few or many cycles, without intersaccadic intervals, around all three axes (3–6). If oscillations occur only in the horizontal plane it is called flutter. Opsoclonus can be triggered by both saccadic and non-saccadic eye movements, by eye closure, and can persist in the dark (7). How neural circuits generate these oscillations is not clear. Note that in our patient, there is a diverse range of waveforms, including opsoclonus (quasi-sinusoidal movements), square-wave oscillations, and square-pulse oscillations (Figure 1). Such a diversity of waveforms has been seen before, and it has been proposed that a common mechanism accounts for all the waveforms (8), but no mechanism has yet been proposed that can do so.

Opsoclonus arises in various diseases [including paraneoplastic, parainfectious, toxic-metabolic, and idiopathic causes (9)], thus one mechanism may not explain all forms of opsoclonus. Nonetheless, opsoclonus is often associated with cerebellar disease (10, 11). Saccades are generated by high-gain burst neurons that are gated by dominant, inhibitory neurons in the brainstem, which pause during saccades [omnipause neurons (OPN)]. Thus, Zee and Robinson (12) proposed that any saccadic oscillations without an intervening interval would require that the OPN be shut off.

Autopsy of one of Cogan’s patients with opsoclonus found encephalitis with lymphocytic infiltration chiefly in the hypothalamus, midbrain, and pons (3). However, studies have shown no consistent pathology in the raphe interpositus (site of the OPN) in patients with opsoclonus (13). Although structural imaging of the brain of opsoclonus patients has not shown any consistent abnormalities, functional imaging has been linked to increased activity in the fastigial nuclei (14). Oguro et al. (15) recorded SPECT images in two patients with opsoclonus and found hyperperfusion in the midline cerebellum (CB) in one, and hypoperfusion in the other patient.

At least three hypotheses for the pathomechanism of opsoclonus/flutter have been proposed, based on different clinical and experimental observations. One line of evidence would suggest a dysfunction of the cerebellar Purkinje cells (PuC). Mutant mice with a modified glutamate receptor on PuC show clustered PuC action potentials, likely induced by climbing fiber activation, and opsoclonus-like eye movements (16). Jen et al. (17) found antibodies to PuC in patients with opsoclonus. They proposed that those antibodies blocked the parallel fiber (PF) input to PuC, allowing spontaneous oscillations generated in the inferior olives (IO) to be passed to the oculomotor vermis through the flocculus.

Alternatively, disinhibition of caudal fastigial nuclei (cFN) might induce unwanted saccades through excitatory projections to brainstem burst neurons (6, 14). Wong et al. (6) modified a lumped model of the saccadic system by adding a negative feedback path from a high pass filtered efference copy of eye position, through the ipsilateral cFN, to the motor error comparator. When the delay and the gain of this feedback pathway were increased (to 20 ms and ~8.5, respectively) to simulate disinhibition of the cFN, ~15 Hz sinusoidal oscillations occurred. However, this mechanism cannot explain how the brain generates non-sinusoidal saccadic oscillations, such as square waves, square pulses, and spindles. This mechanism also does not suggest how a disease process could change the loop’s delay by 20 ms, or raise its gain from normal (~0.7) to ~8, or how it could hold off the OPN, which prevent saccades. Furthermore, lesions of the vermis that disinhibit the cFN cause hypometric saccades, not oscillations (18, 19).

A third idea is that reduction of glycinergic inhibition generates oscillations in the positive feedback loop between left and right saccadic brainstem inhibitory burst neurons (20). This gives rise to small, sinusoidal oscillations, but alone would not be enough to simulate all the waveforms seen in opsoclonus. Thus, none of these theories have been fully supported by lesion studies in animals, clinical findings, or model simulations (21).

Unfortunately, high temporal resolution recordings of eye movements from patients with opsoclonus/flutter, analysis of which could clarify the underlying mechanisms, are extremely rare, because of the difficulty in recording them (given the severity of the clinical symptoms and/or the inability to calibrate the recordings). In our previous study (2), a detailed analysis of one patient’s movements (Figure 1) suggested that ocular oscillations might be generated by a dysfunction of the cerebellar–olivary–brainstem network.

That patient had taken anabolic–androgenic steroids (AAS), which are allosteric modulators of GABA_A_ receptors (GABA_A_R), with both acute and chronic effects. These effects can enhance or diminish the sensitivity of the chloride channel to γ-aminobutyric acid (GABA), depending on the receptor’s subunit composition and the GABA concentration at the synaptic level (22, 23). We shall assume that in our patient, the AAS increased the gain of the chloride current in the GABA_A_R (24). Indeed, the key point of our model is that opsoclonus requires increased inhibition of oculomotor vermis (OMV), cFN, and OPN. Increased inhibition of cFN is not necessarily incompatible with the finding of increased BOLD or fMRI signal of cFN during opsoclonus (14). Increasing cFN inhibition does not necessarily mean that they fire less. As we will see below, in our model the main effect of increasing cFN inhibition is to delay its burst onset, which could be tested in animal models. Thus, even if cFN excitability were significantly reduced, the intense saccadic activity during opsoclonus oscillations (averaged over time) would increase the blood flow and oxygen consumption of the cFN. Our hypothesis of inhibitory receptor dysfunction is very different from prior models, wherein the hypothesis was either damage to the OPN, or damage to the CB causing hyperactivation of the cFN. This is also consistent with the lack of evidence of damage to the CB and pons in opsoclonus. Here, we present model simulations of the cerebellar–olivary–brainstem network that support our hypothesis.

## Subjects and Methods

Two patients described in our previous article (2) developed opsoclonus/flutter after self-administration of performance-enhancing substances. This study was carried out in accordance with the recommendations of the ethics committee of the University of Siena, Italy. All subjects gave written informed consent in accordance with the Declaration of Helsinki. The ethics committee of the University of Siena, Italy, approved this study. Briefly, neuro-ophthalmological examinations revealed horizontal saccadic intrusions and intermittent ocular flutter. Both patients’ brain MRI were normal. CSF showed few oligoclonal bands. Common infectious, toxic, paraneoplastic, and metabolic causes of opsoclonus/flutter were excluded by negative blood and CSF exams. No tumors were found.

Both patients reported a few months of abuse of substances to improve performance. Patients stopped using the drugs after the symptoms began. Patient 1 provided a sample of the compound for testing, which led to the identification of the AAS nandrolone, stanozolol, and testosterone propionate. Treatment with intravenous IgG and benzodiazepine led to recovery in 3–4 weeks in both patients.

### Eye Movement Recording

Eye movement recording during fixation, horizontal (10 and 18° amplitudes), and vertical (8°) saccades was possible in patient 2 because he had inter-oscillatory intervals of steady fixation that allowed a satisfactory calibration. Eye position was recorded at 240 Hz with an ASL 504 eye-tracker device (Applied Science Laboratories, Bedford, MA, USA). All analyses were performed with custom Matlab (The Mathworks, Natick, MA, USA) scripts.

### Model

**Figure 2:**
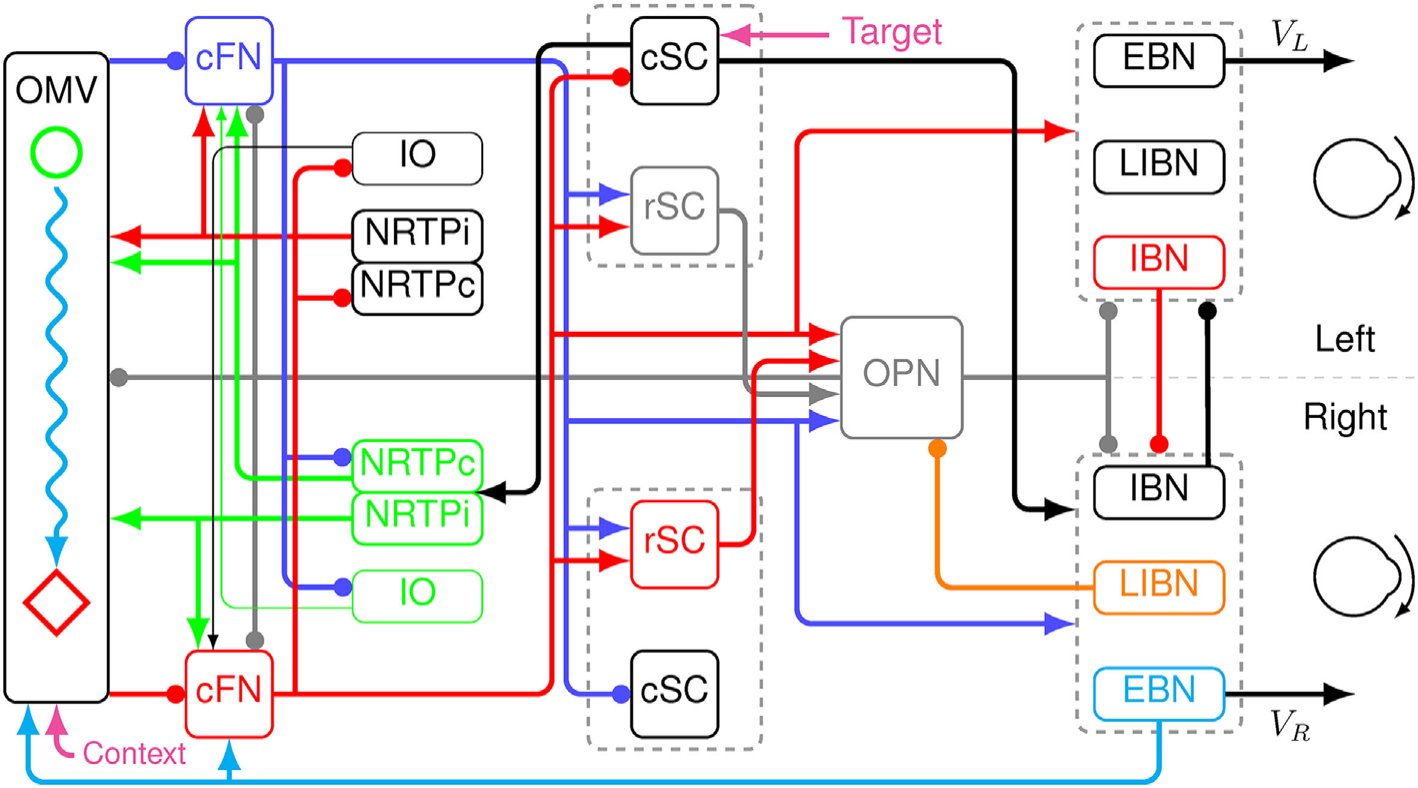
Schematic of a neuromimetic model of the saccadic system, showing the connections needed to make a rightward eye movement. Before the movement, the oculomotor vermis (OMV), the cFN, both rostral SC (rSC) and omnipause neurons (OPN) are on (gray lines), and the premotor burst neurons (PBN) are off. At the start of a rightward saccade, cerebral cortex sends target information to the left caudal SC (cSC) (magenta arrow). The left cSC begins to fire (black line), which inhibits the rSC and excites the right excitatory burst neurons (EBN), inhibitory burst neurons (IBN), and long-lead inhibitory burst neurons (LIBN). However, the OPN are holding the EBN and IBN off. The right LIBN, however, are not held off, and they inhibit the OPN (orange line), allowing the right EBN and IBN to fire, which starts the movement. The left cSC also drives the right nucleus reticularis tegmenti pontis (NRTP), which inhibits the left OMV (at a locus determined by context, magenta arrow) and excites both cFN (green lines). The left cFN begins driving the right PBN (blue lines). As the movement proceeds, an efference copy of eye velocity is fed back from the right EBN to the OMV (cyan line). This causes a wave of inhibition to spread to the right across the OMV (cyan wavy line). Connectivity in the vermis must be left–right symmetric, because it must make saccades in both directions. The spread is not symmetric because the feedback signal is a rightward velocity, which causes the spread to be to the right only. When the wave of inhibition reaches the location corresponding to the ending point of the saccade on the right side of the OMV (red diamond), the right cFN is disinhibited (red line). The right cFN excites the left PBN overcoming the inhibition by the right PBN. The left IBN comes on and inhibits the right PBN, choking off the drive to the right motor neurons and stopping the movement. The OPN and rSC reactivate, because of the right cFN input, preventing saccadic oscillations and holding the eyes on target.

A new model was created by extending the saccadic system model used to simulate eye movement disorders in a cerebellar patient (25). The basic architecture is an *adaptive, velocity feedback, integral controller, but without a motor error comparator* (Figure 2). For simplicity, only the horizontal direction of saccades is modeled. Both the superior colliculi (SCs) and the caudal fastigial nuclei (cFN) drive the brainstem premotor burst neurons [PBN; consisting of the short-lead excitatory burst neurons (EBN), inhibitory burst neurons (IBN), and long-lead inhibitory burst neurons (LIBN)]. An efference copy of eye velocity (from the EBN) is fed back to the OMV, which projects to the fastigial oculomotor region in the cFN, closing the loop. A pause in activity starts at a locus in the contraversive (with respect to the saccade direction, e.g., left side for a rightward saccade) OMV corresponding to the amount of drive that the cFN should provide for an individual saccade, based on the context of the movement. If the saccade does not get on target, the CB learns to initiate the pause at a different locus under the same context the next time (26–28). At saccade start, the OMV activity at the initial locus pauses, releasing the contraversive cFN from inhibition. The cFN then fires, exciting the ipsiversive EBN and IBN. During the saccade, a wave of inhibition (driven by feedback of a velocity efference copy from the EBN) spreads across the OMV. Thus, the OMV acts as a spatial integrator of eye velocity. When the inhibition spreads to the ipsiversive OMV, it disinhibits the ipsiversive cFN, which activates the contraversive IBN. The contraversive IBN then inhibits the ipsiversive EBN and IBN. This stops the movement. At this point, the excitation from the ipsiversive cFN also reactivates the OPN. The EBN and IBN have a very high gain, thus, without OPN reactivation the saccade would be followed by an oscillation of back-to-back saccades with no intersaccadic interval. The caption to Figure 2 gives a color-coded explanation of how the model makes a saccade. Details of the model are given in the Appendix in Supplementary Material.

Although our main hypothesis is that opsoclonus results from an increased sensitivity of GABA_A_R, here we simulate a lumped model. This is necessary for two reasons. First, we think that the opsoclonus is caused by abnormal levels of activity in a large network, encompassing the CB, IO, and brainstem. Second, to understand the effects of the GABA_A_R dysfunction at the biophysical level, we would need to know the types of subunits that make up the receptor in the diverse types of neurons in the circuit, in particular, their gains and time constants. Unfortunately, these values are unknown. Thus, we implement the suspected changes in GABA_A_R function simply by changing various gains in a lumped model. Details of which gains are changed are given with each of the simulations in the Section “Results.” Model parameters are given in Table 1, and parameters for the different neuron types are given in Table 2.

**Table 1:** Important model parameters.

Parameter	Value	Function (units)	Parameter	Value	Function (units)
SynDel	0.0008	Synaptic delay (s)	NI G	20	Neural integrator gain
VisDelay	0.05	Visual delay (s)	NI Tc	20	Neural integrator time constant (s)
Refrac	0.2	Refractory disable (s)	Plant Te	0.008	Small plant time constant (s)
ErrThr	0.5	Retinal error threshold (°)	SC Gdisfa	2	Disfacilitation gain from cFN
MaxDis	800	Maximum discharge rate (sp/s)	SC Tdisfa	0.025	cFN disfacilitation time constant (s)
BrstDel	0.05	SC burst delay (s)	SC Gx	1	cSC cross-inhibitory gain
IO G_l_	1	IO low pass gain	SC Tx	0.01	cSC cross-inhibitory time constant (s)
IO Tc	0.1	IO low pass time constant (s)	rSC Delay	0.05	Delay time before burst (s)
IO I_u_	5	IO output upper limit	OMV Gp	1	Cerebellar plant model gain
IO I_l_	0.1	IO output lower limit	OMV Tp	0.008	Cerebellar plant model time constant (s)
IO P_W_	0 or 1	IO noise power	OMV T_f_	0.25	OMV fatigue time constant (s)
IO G_W_	1	IO noise gain	OMV G_l_	0.5	OMV fatigue low pass gain
IO T_W_	0.002	IO noise time constant (s)	OMV T_l_	1.5	OMV fatigue low pass time constant (s)
IO F_io_	30	IO sine frequency (Hz)	OMV C_l_	0.5	OMV fatigue clip low
IO G_io_	0.35	IO sine gain	OMV C_u_	1	OMV fatigue clip high
OMV G_f_	0.34	OMV fatigue gain	OMV G_lk_	0.5	OMV leaky integrator gain
LIBN G_rsc_	6	Inhibitory gain from rSC to LIBN	OMV T_lk_	0.5	OMV leaky integrator time constant (s)
OPN G_rsc_	3	Excitatory gain from rSC to OPN	IBN2EBN	1.2	Gain from IBN to EBN
OPN Tone	200	Bias on OPN	IBN2IBN	0.3	Gain from IBN to contra IBN
cFN2EBN	0.5	Gain from cFN to contra EBN	IBN2LIBN	200	Gain from IBN to LIBN
cFN2IBN	50	Gain from cFN to contra IBN	EBN2IBN	0.01	Gain from EBN to ipsi IBN

*LIBN, long-lead inhibitory burst neurons; EBN, excitatory burst neurons; IBN, inhibitory burst neurons; OPN, omnipause neurons; IO, inferior olive; SC, superior colliculus; OMV, oculomotor vermis; cSC, caudal SC; rSC, rostral SC*.

**Table 2:** Model parameters for different neuron types.

Parameter	EBN	IBN	LIBN	OPN	cFN	rSC	cSC
Gain	1.000	1.000	1.000	1.000	1.000	1.000	1.000
Time constant (s)	0.002	0.002	0.003	0.002	0.003	0.010	0.010
Adaptation gain	0.050	0.050	0.000	0.010	0.000	1.000	1.000
Adaptation TC (s)	0.100	0.100	0.010	0.006	0.003	0.006	0.006
Inhibitory gain	4.000	12.000	0.015	1.000	2.000	0.100	15.000
Excitatory gain	1.000	0.800	1.000	1.000	1.000	1.000	3.100
OPN gain	10.000	100.00	0.000	0.000	0.000	1.000	1.000

*EBN, excitatory burst neurons; IBN, inhibitory burst neurons; LIBN, long-lead inhibitory burst neurons; OPN, omnipause neurons; cSC, caudal SC; rSC, rostral SC*.

## Results

### Normal Saccades

This model assumes that microsaccades (<2°) and macrosaccades (≥2°) are made by the same circuit (29–32). It can simulate amplitudes from 0.5 to 50° in both leftward and rightward directions (Figure S1 in Supplementary Material). For amplitudes above 10°, the cortical circuit assumes that the actual goal of the saccade is only 90% of the target jump. Thus, for large saccades, the model undershoots the target but automatically makes corrective saccades to get on target.

**Figure 3:**
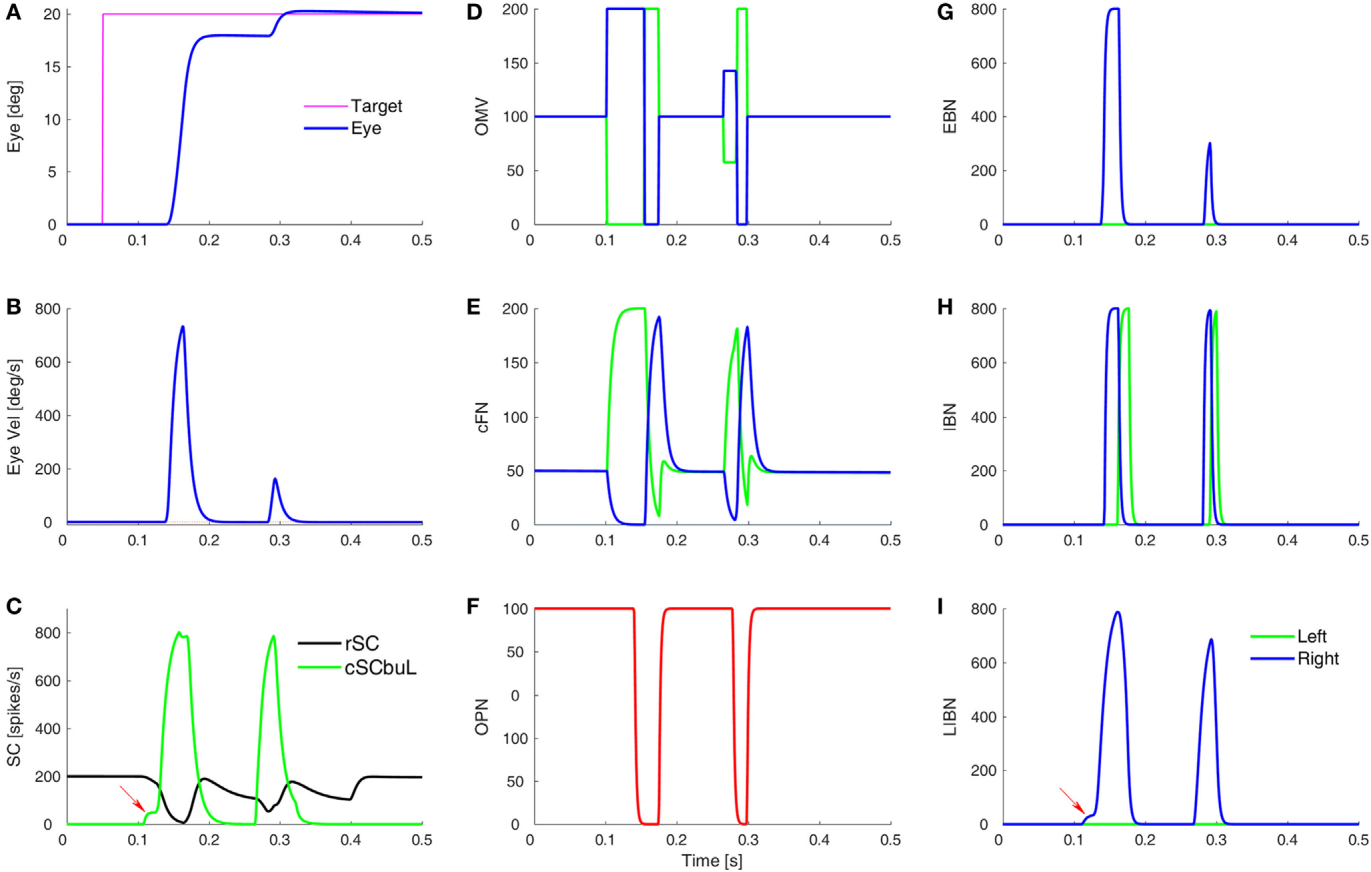
Simulation of a normal saccade to target at 20°, showing time courses of activity in different model areas. **(A,B)** The eye position and velocity traces. **(C)** The activity of burst neurons (BN) in the rostral SC (rSC) and buildup neurons (BUN) in the left caudal SC (cSC). Red arrow indicates the small pre-saccadic buildup of activity (cSC burst neurons are not shown, as they are the same as the buildup neurons, but without the small buildup). **(D)** The activity of the lumped oculomotor vermis (OMV). For a rightward saccade, the left OMV (green) pauses first, followed by the right OMV (blue). **(E)** Activity in the contraversive (green) and ipsiversive (blue) cFN, which are inhibited by their respective OMV. The critical point is that when the ipsiversive cFN (blue) turns on it drives the contraversive inhibitory burst neurons (IBN) on **(H)**. The left IBN’s reactivation stops the saccade. **(F)** Activity of the omnipause neurons (OPN). **(G)** Activity of the excitatory burst neurons (EBN). **(H)** Activity of the IBN. **(I)** The long-lead inhibitory burst neurons (LIBN) are turned on when the cSC BUN activity begins building up (red arrow), because they are not inhibited by the OPN, but the LIBN inhibit the OPN. Ordinate scale for neuronal activity is simulated spikes per second.

A simulated eye movement and the activity in major model neurons are shown in Figure 3. In this highly simplified, one-dimensional, model of the CB, we can see the latency differences between contra- and ipsiversive OMV and cFN bursts (33–35). Indeed, it is our central hypothesis of saccadic system function that the role of the oculomotor vermis is to create this timing difference (26–28). To simulate all three dimensions of opsoclonus (horizontal, vertical, and torsional), the model presented here would have to be duplicated, once for each axis.

### Opsoclonus/Flutter

Opsoclonus/flutter oscillations can exhibit many waveforms. For example, Figure 1 shows eye movements recorded in one session while patient 2 was fixating (abscissa shows time in seconds from the beginning of a single record). Figures 1A–C show examples of the quasi-sinusoidal movements that define classic opsoclonus. Examples of largely horizontal square-wave macrosaccadic oscillations (SWMSO, half-cycles in blue, marked S), a pulse saccadic oscillation without an intersaccadic interval (red, P), and combined half-cycles of SWMSO followed without an intersaccadic interval by a return saccade, which we call a square-pulse oscillation (SP), are shown in Figure 1D. These different waveforms occurred over an interval of just 3 s. We also see an example where the square-pulse changes direction from rightward to leftward (marked −SP). Note that there seems to be a continuum of intersaccadic interval durations (e.g., saccade marked S at about 69.55 s), thus the difference between opsoclonus, square-wave and square-pulse oscillations may only be due to a small change in a few parameters. The vertical component of the eye movement (green) is small but is phase-locked to the horizontal movement. This phase locking is characteristic of opsoclonus. Square-pulse oscillations have been recorded before in patients with opsoclonus, although not commented upon (6, 14).

Figure 1E shows a 2.5 s example of a square-pulse oscillation that grows in amplitude for about 1 s, and then decreases in amplitude [numbers at each extremum indicate gain of the movement, assuming that the eye is trying to get back to the central fixation target (gray line)]. As in Figure 1D, the vertical eye movement is phase locked to the horizontal eye movement. Because of the shape of the movement envelope, these types of oscillations are called *spindles*.

Figure 1 also reveals an asymmetry in the oscillations. In Figures 1D,E, we see that the saccades to the left usually have higher gains than those to the right. We infer from this that the left cFN projections to the right IBN and OPN are weaker than the right cFN projections to the left IBN and OPN, causing a delay in stopping the leftward saccade.

### Tests of Two Prior Hypotheses

Here, we use the new model to test the hypothesis that fastigial disinhibition by Purkinje cell malfunction results in saccadic oscillations, as in the Wong et al. (6) model. In their model, the role of the CB is like that of most models, in that it accelerates the saccade and stops it on target (26, 27, 36). In their simulations, macrosaccadic flutter (back-to-back saccades with no intersaccadic interval) occurs when the loss of OMV inhibition of the cFN increases the gain and the delay in the feedback loop around the brainstem and through the cFN. Their model cannot make other types of oscillations, such as SWMSO, tremor, or square-pulse oscillations. It also faces problems pointed out with the similar Zee and Robinson model (12), in that to make oscillations requires a change in the loop delay that must be set according to the patient’s oscillation frequency, which can cover a very wide range (37).

In the present model, the CB helps accelerate and steer the saccade, and then stops it on target, but it is an adaptive, velocity feedback, integral controller, and not a motor error controller (i.e., its goal is not to reduce the motor error to 0). However, oscillations are controlled by membrane properties of neurons, and not loop delays (37, 38). Our model behaves differently from that of Wong et al. because its OMV and cFN essentially act as a switching network to control the timing of when the ipsiversive and contraversive EBN and IBN turn on and off. Intuition can help us understand a linear feedback controller, but intuition is not helpful in a switching/timing model. Thus, we need simulations to understand the effects of GABA_A_R dysfunction in these models.

**Figure 4:**
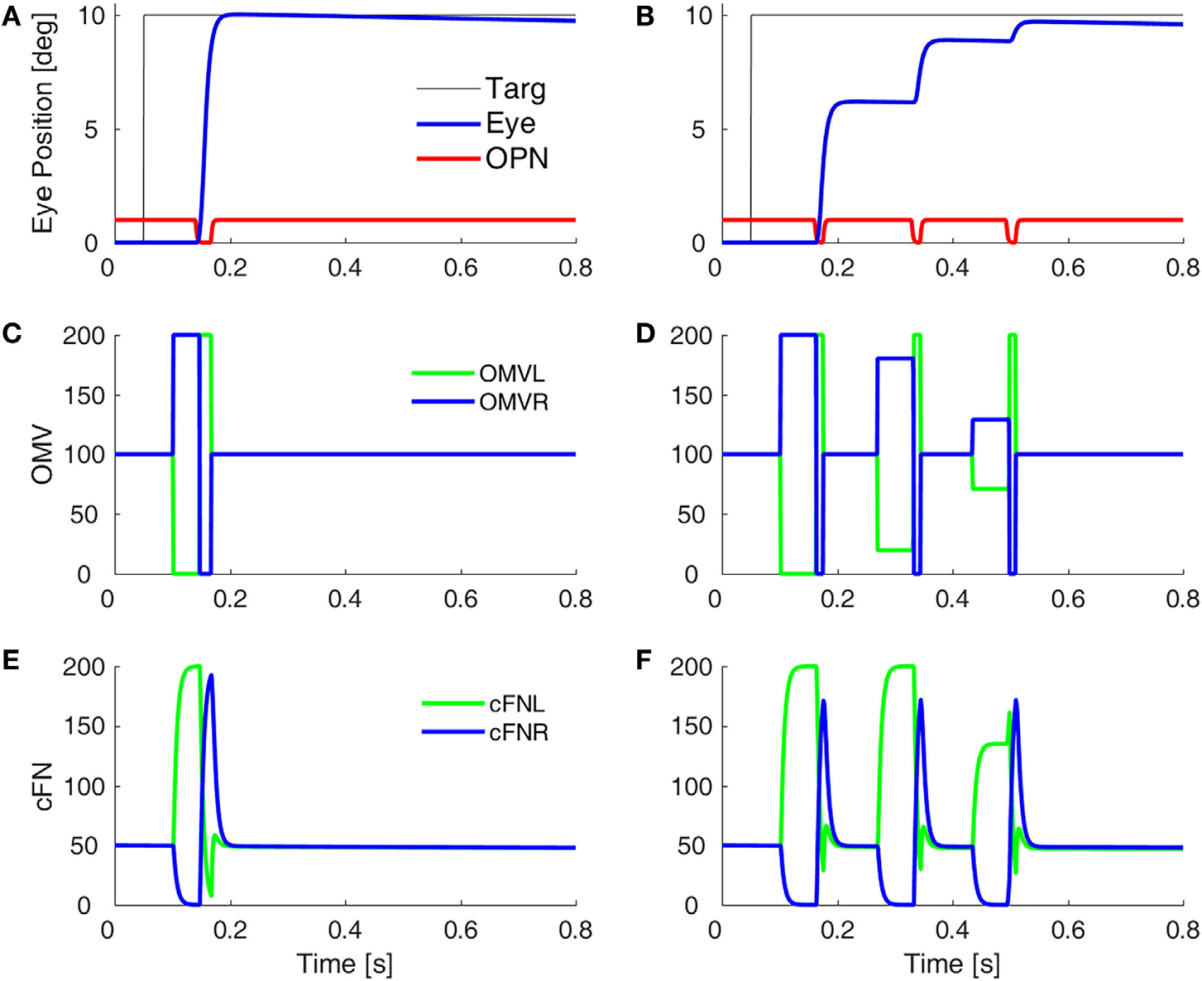
Simulation of loss of vermal inhibition onto fastigial nuclei, i.e., the hypothesis of Wong et al. (6). In our model, loss of oculomotor vermis (OMV) inhibition will cause the ipsiversive cFN to turn on too soon. In the simulation, to get the ipsiversive cFN to start too soon, we increased the gain of the forward model of the plant in the OMV (from 1 to 300), which caused a rapid spread of inhibition from contraversive to ipsiversive, so that the ipsiversive OMV turn off too soon. One might expect this to result in hypermetric saccades, or even oscillations. However, in our model, the OMV play a very different role than in Wong et al.’s model. Importantly, the ipsiversive OMV determines the duration of the saccade. **(A)** Saccade with normal OMV activity. **(B)** Saccade with reduced OMV activity. The saccades are hypometric, because the right cFN is not adequately inhibited and thus reactivates too soon. The OMV activity **(C,D)** and the cFN activity **(E,F)** on the left and right sides.

Before a saccade starts, the locus of initial inhibition in the OMV must be determined (Figure 2, green circle). As the saccade progresses, a wave of inhibition must spread across the OMV (cyan wavy line) until it reaches the ipsiversive side (Figure 2, red diamond), disinhibiting the ipsiversive cFN. Figure 4 shows the effect of making the OMV less active than normal. This will cause the ipsiversive cFN to restart too soon, and the saccade (Figure 4B) will be smaller than normal (Figure 4A). This result contradicts the hypothesis of Wong et al. but is consistent with neurophysiological results, which found that cerebellar vermis lesions result in hypometria, not opsoclonus or sinusoidal oscillations (18, 19).

Another hypothesis of how opsoclonus might be generated suggests that antibodies block PF to PuC synapses, thus reducing inhibition on the flocculus. This allows spontaneous oscillatory activity in the IOs to be passed to the ocular motor nuclei (17). This hypothesis has already been tested in the context of oculopalatal tremor (OPT) (39, 40). OPT has waveforms that look like large, random oscillations, but the movements around each axis are independent. These studies showed that if oscillatory activity from a normal IO projected through the flocculus to the brainstem, the resulting eye movements were very small and had pulsatile waveforms. The development of OPT required a pulsatile oscillator (caused by abnormally tight electrotonic coupling in the IO), and a learned response from the cerebellar cortex to enhance the movement’s gain. Thus, these results suggest that IO oscillations would not, alone, be enough to cause saccadic oscillations in opsoclonus.

### Square-Wave Macrosaccadic Oscillations

Figure 1D shows an example of SWMSO in our patient (marked S). SWMSO and spindles also occur in cerebellar disorders (41). Thus, we look for the effects of an increased gain of GABA_A_R in the CB as the mechanism for causing SWMSO. In the Wong et al. (6) model, the cFN, but not the OMV, is inside the feedback loop, so loss of OMV inhibition to the cFN causes the loop gain to increase. They also had to significantly increase the loop delay (no mechanism for which was proposed). In our model, the behavior is very different, because both the cFN and the OMV are inside the feedback loop and the GABA_A_R dysfunction results in an increased inhibition in both structures.

**Figure 5:**
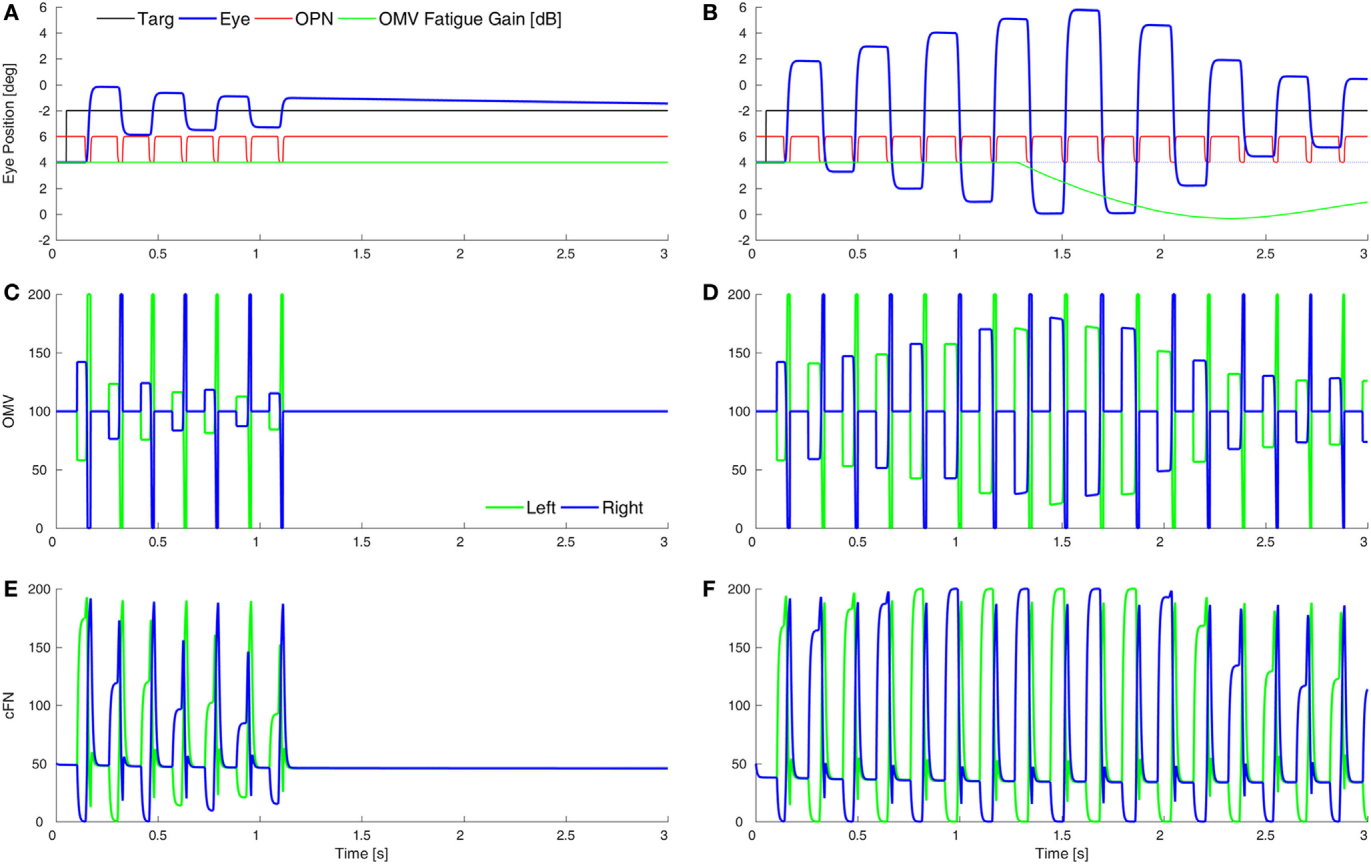
Simulation of square-wave macrosaccadic oscillations (SWMSO). **(A)** A saccade to a 2° target jump. To simulate an increase of inhibition at the GABA_A_ receptors (GABA_A_R) in the oculomotor vermis (OMV), the cerebellar forward plant model gain was reduced from 1 to 0.075, as if the reduction in γ-aminobutyric acid (GABA) inhibition of Purkinje cells was less pronounced, and thus the spread of inhibition across the OMV was slower than normal. The inhibitory gain from the OMV to the cFN was increased from 2.0 to 2.01. The gain of the resultant saccade’s amplitude is almost twice the target amplitude, causing SWMSO. During the oscillation, the gain of the cerebellar fatigue circuit drops very slightly (green line). The oscillations decay because the loop gain is less than 2.0. **(B)** The OMV and cFN GABA_A_R gains are increased even more (represented as a forward plant gain of 0.025, and a cFN inhibitory gain of 2.12), so that the saccadic gain becomes greater than 2. This results in growing oscillations. However, neuronal fatigue (green line) decreases the gain, damping the oscillation, resulting in an SWMSO spindle. **(C,D)** The activity in the simulated OMV and **(E,F)** the activity in the cFN.

Purkinje cells are GABAergic, and we assume that increasing the gain of GABA_A_R in the OMV would mitigate the loss of GABA from the PuC when they become inhibited at saccade start. This would have the effect of slowing the spread of inhibition during the saccade. Thus, to simulate SWMSO, we reduced the gain of the feedback integration within the OMV (Figure 5A). Here, the saccade gains are less than twice normal size, so they decay in size as the oscillation progresses. Also shown is the gain of the fatigue circuit in the CB, which reduces the gain if neuronal activity is too high for too long. In this case, the fatigue gain declines only slightly during the oscillation (green line).

Figure 5B shows the effect of lowering the OMV feedback gain further, which makes the saccade gain higher, and the circuit unstable. The saccade gain is more than twice normal size, and the oscillations grow for a few saccades. However, the high rate of firing and high frequency of saccades result in marked fatigue of the cerebellar activity (green line), and the saccades begin to shrink. The envelope of the oscillation looks like a spindle.

Although the cFN activity is delayed relative to normal during each saccade, the cFN activity over the whole oscillation is very high. This is consistent with findings from imaging studies that the midline CB (Figures 5C,D) and deep cerebellar nuclei (Figures 5E,F) are strongly activated during opsoclonus (14, 15).

It is important to note the difference between the results in Figure 4 (hypometria) and Figure 5 (hypermetria). In Figure 4, we *increased* the gain of the OMV’s forward model of the plant (from 1 to 300), which caused a rapid spread of inhibition from contraversive to ipsiversive. This caused the ipsiversive OMV to turn off too soon (removal of OMV inhibition is Wong et al.’s hypothesis), allowing the ipsiversive cFN to turn on too soon, resulting in hypometria. In Figure 5, we *decreased* the forward model’s gain to represent increased GABA_A_R activity in OMV, thus slowing the spread of inhibition across the OMV and resulting in hypermetria.

### Square-Pulse Macrosaccadic Oscillations and Ocular Flutter

**Figure 6:**
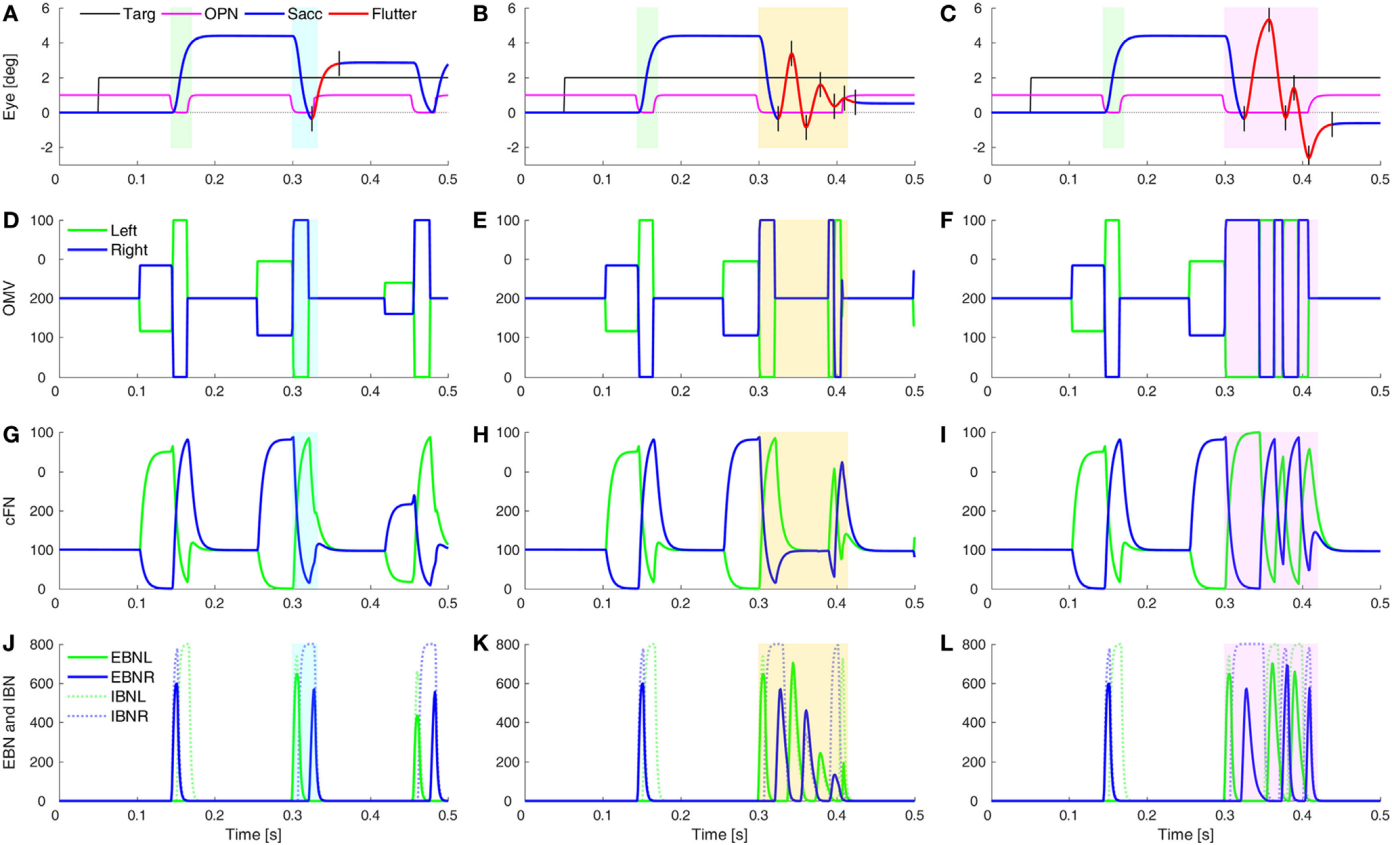
Simulation of post-saccadic oscillations (flutter). Saccades were made hypermetric by changes as in Figure 5. In addition, the reactivation of omnipause neurons (OPN) (magenta) was delayed for leftward saccades (blue rectangles), but not rightward saccades (green rectangles). **(A)** The effect of a slower reactivation of OPN (magenta, delay increased from 10 to 22 ms) resulted in a half-cycle of large amplitude ocular flutter (back-to-back saccades with no intersaccadic intervals) after the leftward saccade (black tick marks and red trace). **(B)** If the reactivation of OPN is delayed even more (to 100 ms), more cycles (here, three half-cycles) of ocular flutter occur. The cerebellum (CB) has become quiescent, so the flutter is driven only by post-inhibitory rebound in the brainstem. **(C)** Similar to panel **(B)**, but the CB was not inhibited during the oscillations, so the oculomotor vermis (OMV) and cFN also contributed to the flutter, making it larger than the flutter in panel **(B)**. **(D–F)** The activity in the simulated OMV, **(G–I)** the activity in the cFN, and **(J–L)** the activity in the excitatory burst neurons (EBN) and inhibitory burst neurons (IBN).

One of the unusual waveforms found in our patient is the square-pulse oscillation (SP in Figure 1D). To simulate this waveform requires two sets of changes. First, the saccades must be hypermetric (as in Figure 5). In addition, it is necessary to delay the onset of the OPN in one direction (here, after leftward saccades). Thus, a rightward movement results in a hypermetric saccade that is followed by a hypermetric leftward saccade. However, after the leftward saccade, the OPN reactivation is delayed, resulting in a return movement with no intersaccadic interval, driven by post-inhibitory rebound (PIR) in the brainstem EBN. Importantly, PIR of the EBN must last at least as long as the return pulse, making the adaptation time constant, *T_a_*, an important parameter (Figure S2 in Supplementary Material). A short delay of OPN reactivation (magenta) allows for a half-cycle (tick marks and red part of trace) of ocular flutter (Figure 6A). If the reactivation of the OPN is further delayed, three half-cycles of flutter can be obtained (Figure 6B). In addition, if the CB does not shut down after the saccade to the left, the OMV and cFN can participate in the oscillation, increasing its amplitude (Figure 6C and compare Figures 6E,F). Thus, random fluctuations in the delay until reactivation of the OPN, and whether or not there is cerebellar involvement, can account for the varying size and number of saccadic pulse waveforms in opsoclonus. Experimental studies will be needed to determine whether EBN PIR and oscillations in the CB contribute to ocular flutter.

What might cause the delay of the OPN reactivation? Under our hypothesis, the opsoclonus is caused by an abnormally high GABA_A_R gain. If the cFN were abnormally inhibited, saccades would be hypermetric, because the ipsiversive cFN would not turn on in time to stop the saccade on target. They might also be too weak to turn the OPN back on at the end of the saccade. OPN receive both GABA and Gly inhibitory transmitters (42). If their GABA_A_R currents were also enhanced, it might take more excitation to reactivate the OPN, causing them to turn on late. This is consistent with the inference from Figure 1 that the left cFN is weaker than the right cFN.

If the OPN reactivation is delayed even more, as may happen during blinks or large off-vertical saccades (37), ocular flutter (back-to-back saccades with no intersaccadic interval) occurs (as in Figure 6B). Pathological flutter has been associated with lesions in the region of the OPN, and with lesions of the projection from cFN to the brainstem (43, 44). Here, we see that the functional effect that causes flutter is the delay in reactivation of the OPN.

**Figure 7:**
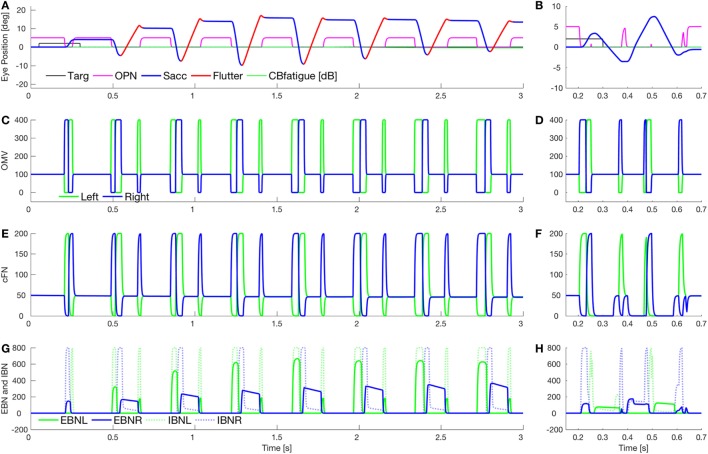
Simulation of square-pulse spindle and opsoclonus. **(A)** Increasing omnipause neurons (OPN) (magenta) reactivation time (to 134 ms) after leftward saccades, and increasing the excitatory burst neurons (EBN) adaptation gain (from 0.05 to 0.01) and time constant (from 100 to 500 ms) gives a spindle-shaped square-pulse waveform with a small dynamic overshoot, like the movement in Figure 1E. The first rightward saccade and all the leftward saccades were visually guided (blue), but with a higher than normal gain [oculomotor vermis (OMV) feedback gain reduced from 1.0 to 0.5, making saccadic gain about 2.2]. After each leftward saccade, a half-cycle of flutter to the right (red) brought the eye back but ended beyond the target. The effect combination of higher gain and fatigue (as in Figure 5B) causes the spindle shape. **(B)** Simulation of a true opsoclonus waveform, similar to that in Figure 1B. The delay of OPN reactivation was increased to 100 ms after both leftward and rightward saccades. Note that neither in the patient nor in the simulation is the waveform a pure sinusoid. This suggests that opsoclonus is actually caused by a series of back-to-back saccades. As OPN recovery time varies, the exact shape of the waveform will vary from quasi-sinusoidal to square wave. **(C,D)** Activity in the simulated OMV. **(E,F)** Activity in the cFN. **(G,H)** Activity in the EBN and inhibitory burst neurons (IBN). Note the pulse part of the waveform is caused by post-inhibitory rebound of the EBN [right only in panel **(G)**, both left and right in panel **(H)**].

Figure 7 shows a combination of the effect of lowering cerebellar feedback gain and fatigue (as in Figure 5) and slowing OPN reactivation (as in Figure 6). This results in a square-pulse oscillation spindle (Figure 7A, like the one in Figure 1E). Here, the OPN are only delayed after leftward saccades. In Figure 7B, we simulate a classic opsoclonus oscillation by delaying OPN after both leftward and rightward saccades. This simulation looks very much like the patient’s eye movement in Figure 1B. The patient’s quasi-sinusoidal oscillations were usually short (0.5–2.5 cycles), with amplitude about 5–10°. There were very few of these movements, compared to square-pulse oscillations. Importantly, these waveforms are not pure sinusoids. They appear to be back-to-back saccades, which is how they were simulated.

## Discussion

The purpose of this model is to show the possible interactions between brain circuits that determine eye movement waveforms. We found that it is not the absolute level of activity, but the relative timing of different areas in the brain, which is important. Here, although the activities in the lumped neurons have highly simplified waveforms, they have the correct timing.

The main predictions of this model are that opsoclonus saccadic waveforms can result from increased GABA inhibition of neurons in the vermis, fastigial nuclei, and brainstem (omnipause and burst neurons), due to increased sensitivity of GABA_A_R.

### GABA_A_R Mechanisms

The GABA_A_R consists of five subunits with many subtypes (α_1–6_, β_1–3_, γ_1–3_, δ, ε, π, θ, and ρ_1–3_) providing diverse receptor functions (45, 46). AAS are allosteric modulators of the GABA_A_R. Their modulatory activity depends upon which steroid is administered and the GABA_A_R subunit composition, being greater for the α_2_ than the α_1_ subunit, but also acting through the δ and ε subunits (22, 24, 47). Subunit expression is different in different brain areas but is poorly defined because of the lack of specificity of markers and the inattention to cell types important for eye movements, e.g., OPN. IO dendrites contain the α_2_ subunit, but their somas contain α_3_ subunits (23, 48, 49). The α_2_ subunit is more prominent than the α_1_ subunit in the cerebellar granule cell and molecular layers (50). Purkinje cells and brainstem reticular formation express the α_1_ subunit, and deep cerebellar nuclei express both subunits (45, 51). We assume that increasing GABA_A_R modulation and decreasing excitation from cFN to OPN would delay OPN reactivation. Despite these speculations, exactly how AAS affected the GABA_A_R in our patients cannot be known. A more detailed biophysical model of opsoclonus awaits further experiments on the effects of GABA_A_R dysfunction in identified cell types.

We have shown how our hypothesis that GABA_A_R are dysfunctional in a cerebellar–olivary–brainstem network (2) can be implemented by varying parameters in the model. Here, we focused on dysfunction that resulted in a higher gain of GABA_A_R. Opsoclonus has many causes, and so other mechanisms besides GABA_A_R modulation may also cause the diverse types of opsoclonus seen in other patients. Whatever the underlying biophysical mechanism, our model allows us to hypothesize how different regions in the brain must be affected to obtain the various waveforms observed in opsoclonus.

### Opsoclonus and Oscillatory Eye Movements

Given the large differences in waveforms seen in patients with opsoclonus, including quasi-sinusoidal, square-wave and square-pulse waveforms, it is not surprising that they have been regarded as different types of movements. However, we have shown that a single model can simulate all of these types of movements, simply by changing a few parameters. Thus, we agree with the hypothesis of Ellenberger et al. (8), who emphasized that these waveforms occur together in the same patients, and thus might be unified as dyskinesias of the saccadic system. Furthermore, although the classic waveform for opsoclonus is a large, sinusoidal oscillation, these may, in fact, simply be back-to-back saccades with no intervening interval, i.e., quasi-sinusoidal oscillations. With low bandwidth recordings (e.g., from electroculograms) these waveforms would be low pass filtered and thus would look sinusoidal. However, higher quality recordings from video or eye coil systems reveal their quasi-sinusoidal nature.

These waveforms can all be obtained from a model of the saccadic system by making appropriate parameter changes in both cerebellar and brainstem circuits, but not by making changes in either alone. We infer from our model that the *mixture* of opsoclonus (quasi-sinusoidal), square-wave and square-pulse oscillations resulting from cerebellar/brainstem dysfunction may commonly co-occur in patients with opsoclonus. Thus, as earlier studies have argued, opsoclonus is not caused by a cerebellar deficit alone. Nor is it caused by lesions of the OPN region. Instead, we hypothesize that opsoclonus occurs when neuronal activity in the CB and brainstem are mistimed.

## Ethics Statement

This study was carried out in accordance with the recommendations of the ethics committee of the University of Siena, Italy. All subjects gave written informed consent in accordance with the Declaration of Helsinki. The ethics committee of the University of Siena, Italy, approved this study.

## Author Contributions

EP proposed the underlying theory. LO implemented the model and ran the simulations. Both authors wrote the manuscript.

## Conflict of Interest Statement

The authors declare that the research was conducted in the absence of any commercial or financial relationships that could be construed as a potential conflict of interest.

## Supplementary Material

Figure S1 Family of saccades simulated with normal parameter values. **(A)** Saccades from −50 to +50°. Note that saccades larger than 10° undershoot the target by 10%. The model automatically makes a corrective saccade that gets on target (gray lines). **(B)** Inset magnifies saccades to left and right 0.5 and 2° targets. **(C)** Velocity traces for saccades in panels **(A,B)**. Peak speeds were approximately matched to those found by Clark and Stark (52) in normal human subjects.

Figure S2 Simulation of post-inhibitory rebound (PIR) in a model neuron (Eq. 1). PIR depends heavily on three parameters: the gain and time constant of the adaptation element, and the amount of hyperpolarization before the rebound. Here, the excitatory input was set to 0. The neuron has two inhibitory inputs, one for γ-aminobutyric acid (GABA) and one for glycine [NB: the omnipause neurons (OPN) and the IBN are glycinergic, and some LIBN are GABAergic]. The total inhibition to the neuron is thus the sum of the GABA and Gly inputs. For convenience, the sum is set to 1. GABAergic inhibitory input changed briefly from 0.8 to 0. Glycinergic OPN inhibition changed briefly from 0.2 to 0. Thus, the total inhibition to the cell changed from 1.0 to 0. **(A)** Effect of changing the adaptation gain (*G_**a**_*) on the peak of the rebound activity (adaptive time constant was set to 6 ms). **(B)** Effect of changing the adaptive time constant (*T_**a**_*, with adaptive gain set to 1.0). Other parameters for the neuron were: *G_**l**_* = 1, *T_**l**_* = 2 ms, *G_**o**_* = 1.0, δ = 0.8 ms. As the time constant increases from 2 to 21 ms, the amplitude and width of the rebound activity increase. Above 21 ms, however, the amplitude begins to decrease. The duration of the rebound is truncated when the OPN resume firing. **(C)** Effect of reducing γ-aminobutyric acid (GABA) inhibition on rebound amplitude. The maximum amount of GABA ranged from 0.0 to 0.8 (accounting for from 0 to 80% of the inhibition on the neuron). As the proportion of GABA was reduced, the hyperpolarization decreased, and the rebound amplitude decreased.

## Abbreviations

BN, SC burst neurons; BUN, SC buildup neurons; CB, cerebellum; cFN, caudal fastigial nucleus; cSC, caudal SC; EBN, excitatory burst neurons; GABA, γ-aminobutyric acid; PuC, Purkinje cells (vermis); Glu, glutamate; Gly, glycine; IBN, inhibitory burst neurons; IO, inferior olive; LIBN, long-lead inhibitory burst neurons; NRTP, nucleus reticularis tegmenti pontis; NRTPc, NRTP → contra OMV; NRTPi, NRTP → ipsi OMV; PF, parallel fiber; OMV, oculomotor vermis; OPN, omnipause neurons; PIR, post-inhibitory rebound; rSC, rostral SC; SC, superior colliculus; SWMSO, square-wave macrosaccadic oscillations; FOR, fastigial oculomotor region.
