# Supplementary material for: A GABAergic Dysfunction in the Olivary–Cerebellar–Brainstem Network May Cause Eye Oscillations and Body Tremor. II. Model Simulations of Saccadic Eye Oscillations
Source: Front Neurol. 2017 Aug 4;8:372. doi: 10.3389/fneur.2017.00372 (PMC5543285; doi:10.3389/fneur.2017.00372)
Supplement: Supplementary file 1 [file Data_Sheet_1.PDF]

Appendix

Lumped Neuron with Post-Inhibitory Rebound

In simulations of saccades, we used a lumped model, with a simplified IO. This eliminated the noise normally contributed by the IO, allowing the model's simulation runs to be compared more easily. Anatomical regions of the brain were simulated with lumped representations of neurons, e.g., there were only two EBN neurons, one on the left and one on the right. All neurons were represented as adaptive low pass filters, with post-inhibitory rebound (PIR) (Ramat et al., 2005).

Although the presence of PIR in brain stem neurons has not been studied, it is well known to exist in other neurons, for example in the deep cerebellar nuclei (Aizenman and Linden, 1999; Alvina et al., 2008; Alvina et al., 2009; Zheng and Raman, 2011; Reato et al., 2015), nucleus ambiguus (Rekling and Feldman, 1997), medial septal neurons (Brazhnik and Fox, 1997), and nucleus tractus solitarii (Jean, 2001). Thus, as we have stated before (Ramat et al., 2005; Miura and Optican, 2006), we consider the existence of post-inhibitory rebound in ocular motor neurons as a major prediction of the model.

Unfortunately, there are no measurements of the time constant of PIR in brain stem neurons. Making this problem harder is that the time course of the PIR depends upon the level and duration of hyperpolarization, and the levels of cations available for the T-type Ca^{2+} channel. In DCN neurons, the time course estimated from Aizenman and Linden's (1999) examples can range from 55 to 200 ms. This is similar to the range of time constants used in our previous conductance-based model of saccade slowing (Miura and Optican, 2006), and to the range used in these simulations. We shall see in the simulations that long time constants are needed to reproduce square-pulse oscillations.

The transfer function of a neuron is thus:

$$\left\lfloor \left\lfloor 1+\frac{sG_aT_a}{sT_a+1}\right\rfloor \frac{G_l}{sT_l+1}\right\rfloor e^{-s\delta} \quad (1)$$

where s is the Laplace variable. The adaptation factor has gain and time constant G_a and T_a (typically 0.05 – 0.10 and 100 - 200 ms). The low pass filter has gain and time constant G_l and T_l (typically 1.0 and 2 ms). The floor corner brackets (⌊ ⌋) imply that the output is nonnegative. The output is delayed by δ, set to the synaptic delay (0.8 ms). Each neuron has three inputs, one excitatory, one inhibitory, and a dominant inhibition from OPN (with three gains, G_e, G_i and G_o, typically 4, 4 and 10). A simulation of the post-inhibitory rebound is shown in Fig. S2 for various sets of parameters. Parameters for each neuron type are given in Table 2.

Brain Areas

Cortex

The model includes a simple logic circuit to simulate the selection of the target by the frontal eye fields and lateral intraparietal cortex. The retinal error is first delayed by 50 ms. Then, the retinal error is compared against a post-saccadic refractory period (350 ms) and an error threshold (0.5º). If it is time for a new saccade, the value of the saccade goal in retinotopic coordinates is held in a sample-and-hold circuit, which sends it to the other parts of the model. When there has been no change in retinal error, the cortex sends a command to fixate to the rostral SC (rSC).

Superior Colliculus (SC)

The SC is the structure in the model that starts the saccade. The rSC consists of one neuron to represent rSC activity on both sides. In the caudal SC (cSC) there is one buildup neuron (BUN) and one burst neuron (BN) on each side. Between saccades, the rSC is excited by the fixation

command from cortex. This rSC activity inhibits the activity in the cSC neurons on both sides. When the cortical fixation command goes away, rSC activity begins to decay. At the same time, a goal signal is sent to the cSC on the contraversive side (with respect to the movement, e.g., left side for a rightward saccade). The contraversive BUN in the cSC begin firing at a low level. After a delay (50 ms) the contraversive BN and BUN begin to burst, shutting off the rSC. The two cSC weakly inhibit each other, and strongly inhibit the rSC. These mutual inhibitions create a toggle circuit, so that the cSC and the rSC cannot both be active at the same time. The toggle switches activity from rSC to cSC when the fixation command goes off and the saccade goal from cortex provides an input to the contraversive cSC. The reverse toggle, from cSC to rSC, happens when the activity in the cSC runs down because of the disfacilitation from the cFN, and the cFN excites the rSC. The outputs of the cSC go to the rSC, cerebellum, inferior olive and premotor burst neurons.

Cerebellum

The cerebellum has three key roles to play (Lefevre et al., 1998; Quaia et al., 1999; Optican and Quaia, 2002). The first is to determine, based on the context of the movement (e.g., desired retinotopic goal, current eye orientation, target velocity, etc.), where the initial locus of activity should be in the OMV. If the saccade does not get on target, the cerebellum adapts by moving the initial locus to another spot. This learning behavior is not modeled here, as the model is not distributed, but lumped. Instead, the spread across the OMV is represented by a leaky integrator. Activity is initially depressed in the contraversive OMV. The depression of OMV activity on the ipsiversive side occurs when the leaky integrator reaches a threshold.

The second function of the cerebellum is to drive the eye toward the target, steering it if it gets off course. This model incorporates this function by feeding back an efference copy of eye

velocity from the EBN to the OMV. The velocity feedback passes through a forward model of the plant to reach the leaky integrator, to keep the internal signals in temporal and amplitude correspondence with the real eye position.

The third function is to stop the saccade on target. This is accomplished by comparing the leaky integrator output with a saccadic-goal dependent threshold. When the sign of the comparison changes, the ipsiversive OMV are inhibited and the ipsiversive cFN begin firing, which turns on the contraversive IBN, which inhibits the ipsiversive EBN and IBN, stopping the saccade. To keep the EBN and IBN from oscillating, the OPN must turn on and hold the eyes still (Optican and Pretegiani, 2017).

Oculomotor Vermis (OMV)

The OMV must determine where to initiate the locus of inhibition (green circle, Fig. 2) at the beginning of the saccade, and must spread the inhibition across the OMV toward the ipsiversive side (wavy cyan line to the red diamond, Fig. 2). In the brain, there are two main cell types in the OMV, burst and pause neurons (Ohtsuka and Noda, 1995). In our model, we use a single cell type that is a composite: the contraversive neuron pauses for saccade start and bursts for saccade end, and *vice versa* for the ipsiversive neuron. The OMV neurons are represented in our model by a leaky integrator (dumped at the end of every saccade):

$$\frac{0.5}{0.5s + 1} \qquad (2)$$

a directional splitter, and a threshold. The OMV integrates an efference copy of the eye velocity drive, but it does not compare the output to the desired eye displacement, i.e., there is no comparator (and thus no motor error signal) in our model (Optican and Quaia, 2002). When the

integrator output crosses a saccadic-goal dependent threshold, the ipsiversive OMV pause and the ipsiversive cFN begin firing.

The input comes from a forward model of the plant:

$$\frac{1}{sT_p + 1} \tag{3}$$

The output is split into leftward and rightward signals (by half-wave rectifiers), and sent to two neurons representing the left and right OMV (OMVL and OMVR). The OMV inhibits the cFN on the same side. A threshold represents the starting point (the green circle) for the desired saccade amplitude. When the progress of the saccade reaches the midline (as indicated by the leaky integrator matching the threshold in our simulations, e.g., at 4.5° for a 10° saccade) the ipsiversive OMV turns on. This simulates the cyan wavy line and the red diamond in Fig. 2. For example, for a rightward saccade, the OMVL is initially on, but when the leaky integrator reaches the threshold value the OMVR turns on. Note that the threshold value is not the desired saccade amplitude, but depends upon the movement context and the time needed by the circuit to switch its activity and stop the saccade (another reason that this is not a feedback error controller). An off-line simplex algorithm adapted the threshold parameters for saccades from the set ± [0.5, 1, 2, 3, 5, 10, 15, 20, 25, 30, 40, 50, 55, 60°] (Fig. S1). Other amplitudes were interpolated with a shape-preserving piecewise cubic function (Matlab's *pchip* function). The required amplitudes and peak velocities were based on recordings of normal human subjects (Figure 5 in Clark and Stark, 1974). The velocity nonlinearity (presumably due to weights from the SC to the premotor burst neurons) was determined by a weighting factor (V_{nl}) in the premotor burst neurons:

$$V_{nl} = 1138\left(1 - e^{-A/22}\right) \quad (4)$$

where A is the amplitude of the saccade. The cerebellum exhibits fatigue, so that if a very large movement is called for, the output will be less than desired. The fatigue assumes that there is a depletable resource in the neuron, which is replenished at a fixed rate (e.g., like an electrogenic sodium pump). The input is low pass filtered (gain, $G_f = 0.05$, and time constant, $T_f = 250\ ms$) to obtain an estimate of the power in the signal. The output is low pass filtered (gain, $G_l = 1.0$, and time constant, $T_l = 1.0\ s$) and clipped (between $C_l = 0.05$ and $C_u = 1.0$). The effect of this circuit is to form the product of a decaying exponential with the signal, if the signal is too large for too long.

Caudal Fastigial Nuclei (cFN)

The cFN are modeled very simply as single, lumped neurons (eq. 1), one for each side. The cFN neurons receive inhibitory inputs from the ipsilateral OMV and the OPN. The cFN also receive an excitatory bias input from the contralateral IO.

Premotor Burst Neurons (PBN)

Omnipause Neurons (OPN)

A single, lumped neuron (eq. 1) represents all the OPN. It receives excitatory inputs from the rSC, the cFN, and a constant tone (for spontaneous firing). It receives inhibitory inputs from the long-lead inhibitory burst neurons (LIBN) (Scudder et al., 1988; Shinoda et al., 2011), some of which are in the cMRF and are GABAergic (Wang et al., 2013). The output of the OPN goes bilaterally to the premotor burst neurons, EBN and IBN, but not LIBN. The output also goes to

the cerebellum, where it blocks input to the OMV's leaky integrator. A circuit also allows the onset of the OPN to be delayed after saccades to the left or right.

Burst Neurons

The PBN are divided into left and right sides, and there is a single, lumped neuron for each side's EBN, IBN and LIBN. Considering a saccade to the right, the right EBN and IBN receive an excitatory input from the left cSC burst neuron and the left cFN. They receive glycinergic inhibition from the OPN and the left IBN. The right LIBN receive excitatory input from the left cSC buildup neurons, and inhibition from left IBN, LIBN and the rSC, but not from the OPN.

Final Common Path

All the signals that drive the eye movement (EBN and vestibular nuclei) converge on this pathway. We use a simplified plant model with a time constant of 8 ms:

$$\frac{1}{sT_e + 1} \tag{5}$$

The input to the eye plant is then just the output of the neural integrator (NI). The NI is represented by a leaky low pass filter (cf. eq. 2) with a gain of 20 and a time constant of 20 s.

Simulation

The model was simulated in Matlab and Simulink (The Mathworks, Natick, MA), version R2015b. The ODE solver was the third order Bogacki-Shampine method, with a fixed step size of 0.1 ms. Major model parameters are given in Table 1, and neuron parameters are given in Table 2. A family of saccades is shown in Fig. S1. Simulations of post-inhibitory rebound (PIR) for different parameters are shown in Fig. S2. Further details of the simulation are available in the Matlab/Simulink program "Opsoclonus.xls", available from the corresponding author.

References

Aizenman, C.D., and Linden, D.J. (1999). Regulation of the rebound depolarization and spontaneous firing patterns of deep nuclear neurons in slices of rat cerebellum. *J Neurophysiol* 82(4)**,** 1697-1709.

Alvina, K., Ellis-Davies, G., and Khodakhah, K. (2009). T-type calcium channels mediate rebound firing in intact deep cerebellar neurons. *Neuroscience* 158(2)**,** 635-641. doi: 10.1016/j.neuroscience.2008.09.052.

Alvina, K., Walter, J.T., Kohn, A., Ellis-Davies, G., and Khodakhah, K. (2008). Questioning the role of rebound firing in the cerebellum. *Nat Neurosci* 11(11)**,** 1256-1258. doi: 10.1038/nn.2195.

Brazhnik, E.S., and Fox, S.E. (1997). Intracellular recordings from medial septal neurons during hippocampal theta rhythm. *Exp Brain Res* 114(3)**,** 442-453.

Clark, M.R., and Stark, L. (1974). Control of human eye movements: III. Dynamic characteristics of the eye tracking mechanism. *Mathematical Biosciences* 20(3-4)**,** 239-265.

Jean, A. (2001). Brain stem control of swallowing: neuronal network and cellular mechanisms. *Physiol Rev* 81(2)**,** 929-969.

Lefevre, P., Quaia, C., and Optican, L.M. (1998). Distributed model of control of saccades by superior colliculus and cerebellum. *Neural Netw* 11(7-8)**,** 1175-1190.

Miura, K., and Optican, L.M. (2006). Membrane channel properties of premotor excitatory burst neurons may underlie saccade slowing after lesions of omnipause neurons. *J Comput Neurosci* 20(1)**,** 25-41. doi: 10.1007/s10827-006-4258-y.

Ohtsuka, K., and Noda, H. (1995). Discharge properties of Purkinje cells in the oculomotor vermis during visually guided saccades in the macaque monkey. *J Neurophysiol* 74(5)**,** 1828-1840.

Optican, L.M., and Pretegiani, E. (2017). What stops a saccade? *Philos Trans R Soc Lond B Biol Sci* 372(1718)**,** 20160194. doi: 10.1098/rstb.2016.0194.

Optican, L.M., and Quaia, C. (2002). Distributed model of collicular and cerebellar function during saccades. *Ann N Y Acad Sci* 956**,** 164-177.

Quaia, C., Lefevre, P., and Optican, L.M. (1999). Model of the control of saccades by superior colliculus and cerebellum. *J Neurophysiol* 82(2)**,** 999-1018.

Ramat, S., Leigh, R.J., Zee, D.S., and Optican, L.M. (2005). Ocular oscillations generated by coupling of brainstem excitatory and inhibitory saccadic burst neurons. *Exp Brain Res* 160(1)**,** 89-106. doi: 10.1007/s00221-004-1989-8.

Reato, D., Tara, E., and Khodakhah, K. (2015). Deep Cerebellar Nuclei Rebound Firing In Vivo: Much Ado About Almost Nothing? *The Neuronal Codes of the Cerebellum***,** 27-51.

Rekling, J.C., and Feldman, J.L. (1997). Calcium-dependent plateau potentials in rostral ambiguus neurons in the newborn mouse brain stem in vitro. *J Neurophysiol* 78(5)**,** 2483-2492.

Scudder, C.A., Fuchs, A.F., and Langer, T.P. (1988). Characteristics and functional identification of saccadic inhibitory burst neurons in the alert monkey. *J Neurophysiol* 59(5)**,** 1430-1454.

Shinoda, Y., Sugiuchi, Y., Takahashi, M., and Izawa, Y. (2011). Neural substrate for suppression of omnipause neurons at the onset of saccades. *Ann N Y Acad Sci* 1233**,** 100-106. doi: 10.1111/j.1749-6632.2011.06171.x.

Wang, N., Perkins, E., Zhou, L., Warren, S., and May, P.J. (2013). Anatomical evidence that the superior colliculus controls saccades through central mesencephalic reticular formation gating of omnipause neuron activity. *J Neurosci* 33(41)**,** 16285-16296. doi: 10.1523/JNEUROSCI.2726-11.2013.

Zheng, N., and Raman, I.M. (2011). Prolonged postinhibitory rebound firing in the cerebellar nuclei mediated by group I metabotropic glutamate receptor potentiation of L-type calcium currents. *J Neurosci* 31(28)**,** 10283-10292. doi: 10.1523/JNEUROSCI.1834-11.2011.
