# Supplementary figures and images for: A GABAergic Dysfunction in the Olivary–Cerebellar–Brainstem Network May Cause Eye Oscillations and Body Tremor. II. Model Simulations of Saccadic Eye Oscillations
Source: Front Neurol. 2017 Aug 4;8:372. doi: 10.3389/fneur.2017.00372 (PMC5543285; doi:10.3389/fneur.2017.00372)

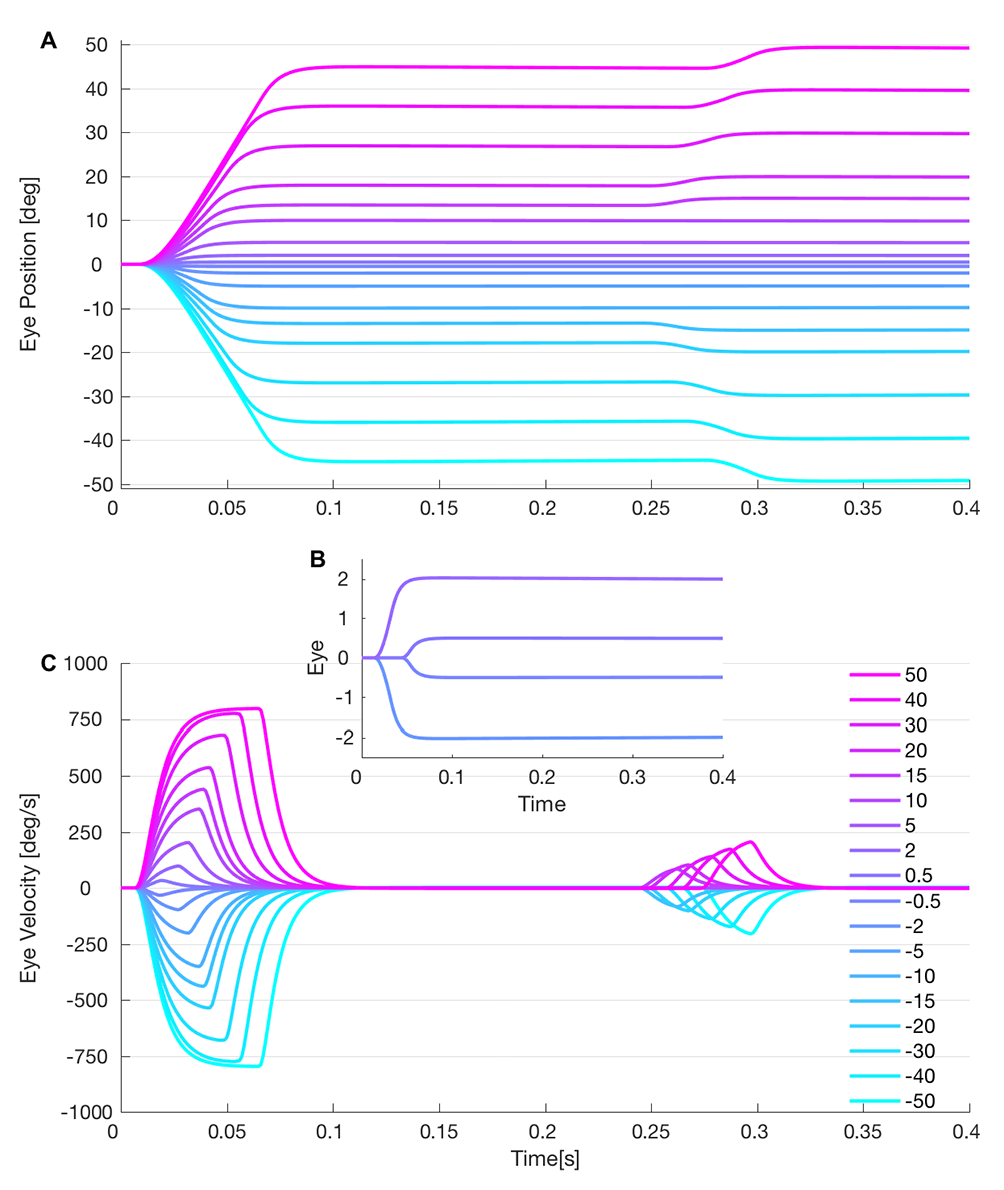

Supplement: Figure S1 — Family of saccades simulated with normal parameter values. (A) Saccades from −50 to +50°. Note that saccades larger than 10° undershoot the target by 10%. The model automatically makes a corrective saccade that gets on target (gray lines). (B) Inset magnifies saccades to left and right 0.5 and 2° targets. (C) Velocity traces for saccades in panels (A,B). Peak speeds were approximately matched to those found by Clark and Stark (52) in normal human subjects. [file Image_1.TIF]

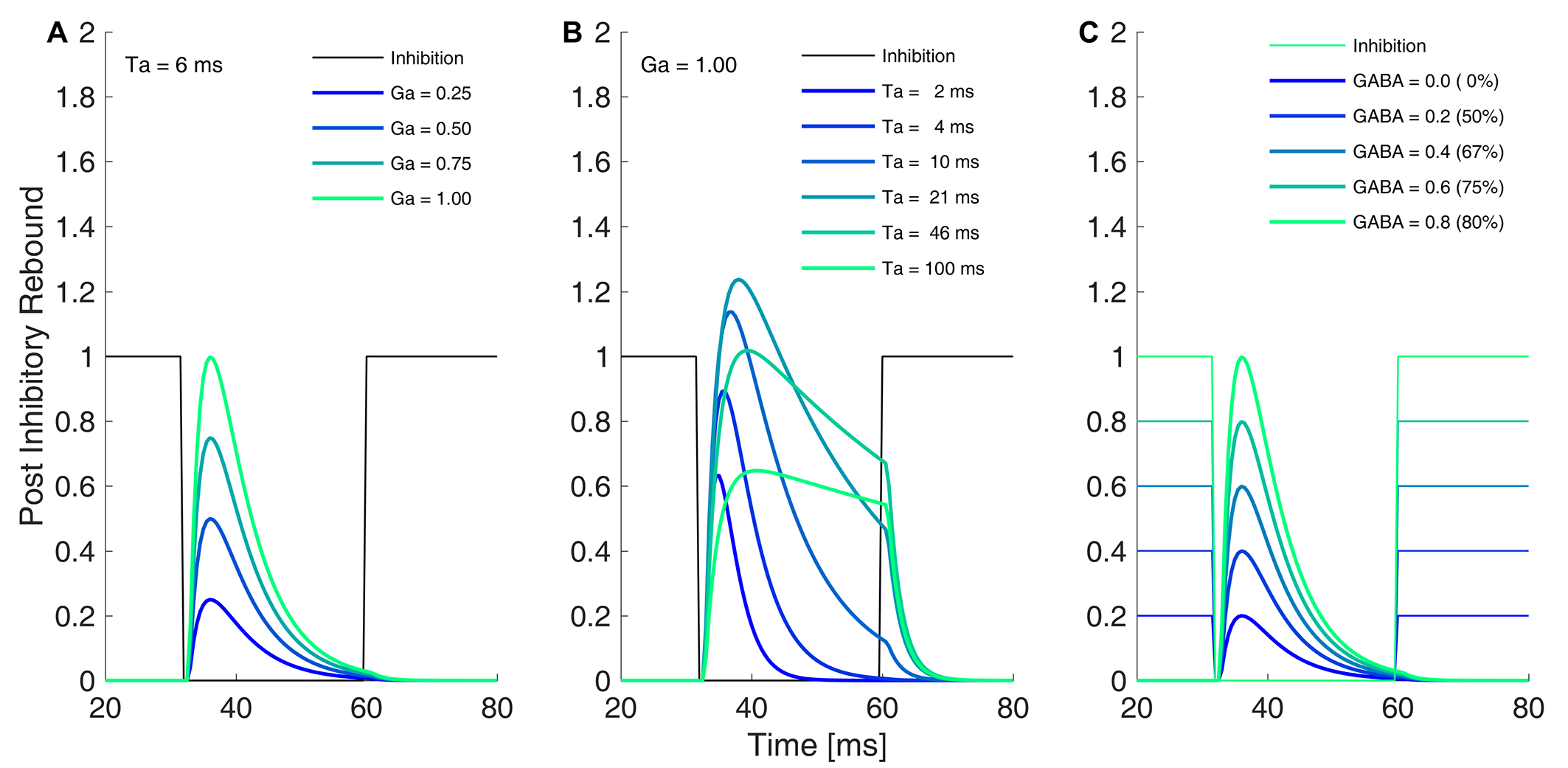

Supplement: Figure S2 — Simulation of post-inhibitory rebound (PIR) in a model neuron (Eq. 1). PIR depends heavily on three parameters: the gain and time constant of the adaptation element, and the amount of hyperpolarization before the rebound. Here, the excitatory input was set to 0. The neuron has two inhibitory inputs, one for γ-aminobutyric acid (GABA) and one for glycine [NB: the omnipause neurons (OPN) and the IBN are glycinergic, and some LIBN are GABAergic]. The total inhibition to the neuron is thus the sum of the GABA and Gly inputs. For convenience, the sum is set to 1. GABAergic inhibitory input changed briefly from 0.8 to 0. Glycinergic OPN inhibition changed briefly from 0.2 to 0. Thus, the total inhibition to the cell changed from 1.0 to 0. (A) Effect of changing the adaptation gain (Ga) on the peak of the rebound activity (adaptive time constant was set to 6 ms). (B) Effect of changing the adaptive time constant (Ta, with adaptive gain set to 1.0). Other parameters for the neuron were: Gl = 1, Tl = 2 ms, Go = 1.0, δ = 0.8 ms. As the time constant increases from 2 to 21 ms, the amplitude and width of the rebound activity increase. Above 21 ms, however, the amplitude begins to decrease. The duration of the rebound is truncated when the OPN resume firing. (C) Effect of reducing γ-aminobutyric acid (GABA) inhibition on rebound amplitude. The maximum amount of GABA ranged from 0.0 to 0.8 (accounting for from 0 to 80% of the inhibition on the neuron). As the proportion of GABA was reduced, the hyperpolarization decreased, and the rebound amplitude decreased. [file Image_2.TIF]
